# Cross-sectional study of adherence to venous thromboembolism prophylaxis guidelines in hospitalized patients. The Trombo-Brit study

**DOI:** 10.1186/1477-9560-10-7

**Published:** 2012-05-18

**Authors:** Federico Jorge Bottaro, José Manuel Ceresetto, John Emery, Julio Bruetman, Nicholas Emery, Débora Pellegrini, Victoria Pinoni, Gastón Piñeiro, Laura Fox, María Eugenia Orrico, Silvina Palmer, Sebastián Prieto, Eduardo Bullorsky

**Affiliations:** 1 Servicio de Clínica Médica, Hospital Británico, Perdriel 74, Buenos Aires 1280, Argentina; 2 Servicio de Hematología, Hospital Británico, Solís 2174, Buenos Aires, 1280 Argentina

## Abstract

**Background:**

DVT is the main cause of death in hospitalized patients and thromboprophylaxis is the only way to prevent these deaths. International recommendations suggested that active monitoring of DVT/PE prophylaxis can improve the efficacy in Hospitals.

**Methods:**

We performed a cohort study in three consecutives periods to evaluate DVT prophylaxis in 388 adults hospitalized in a General Hospital.

**Results:**

85% of the population had high risk factors for DVT. Thromboprophylaxis was in accordance with local and International guidelines (ACCP 2008) in 72.7% and 86% of the patients respectively. No significant difference could be founded between clinical and surgical patients. One every 10 patients received higher prophylaxis than suggested by guidelines and two out of ten received deficient or no prophylaxis. The worst 2 groups of patients were those with moderate/low risk of DVT and the group with a contraindication to pharmacologic prophylaxis. We observed a progressive improvement of the DVT prophylaxis in the 3 periods of evaluation.

**Conclusions:**

Although the rate of recommended thromboprophylaxis is higher than many other reports in the region we still have some areas where we need to improve. Regular audits like these are very helpful to find out what specific areas of the hospital needs some careful attention in order to have a better quality of assistance.

## Introduction

At present, venous thromboembolism (VTE) is a major problem for international public health policies [1]. It is the first cause of death in hospitalized patients, and the third cause of death of cardiovascular origin. In fact, it is the most important clinical problem in many patient subgroups: it is the first cause for readmission after hip arthroplasty and the first cause of death during pregnancy, after gall-bladder surgery and hernia repair [2]. The Federal Agency for Health Research and Quality (AHRQ) in the U.S. (United States) considers that the appropriate use of thromboprophylaxis (TP) is the most important procedure today for an institution to improve the quality of its practices [1].

One of the dominant characteristics of this disease is that for every symptomatic pulmonary embolism diagnosed, there are 2.5 cases of VTE that we are not able to identify. Moreover, 40 to 60% of the deaths from VTE occurs in patients whom lacked a previous diagnosis of deep vein thrombosis (DVT), and 20% of the patients have a sudden death secondary to massive embolism as their first and only symptom [3]. To put these numbers in perspective, the total number of deaths from this condition in hospitalized patients in England surpasses that of the combined deaths from breast cancer, AIDS (acquired immune deficiency syndrome) and traffic accidents [4].

Antithrombotic prophylaxis is the only tool we have to prevent these deaths. For this reason multiple guidelines and consensus have been developed in multiple countries for effective prevention in patients at risk [5].

Nevertheless, despite the efforts of many physicians reflected in innumerable journals, and the seriousness that this problem has merited in the published literature, the truth is that not enough is done for adequate prophylaxis. The ENDORSE study, a cross-sectional study that evaluated the appropriateness of TP indications according international antithrombotic guidelines done in 68,000 hospitalized patients worldwide (including four Latin American countries), showed that only 50% of patients received adequate TP [6]. Furthermore, 36% of surgical patients and 52% of medical patients did not receive any prophylaxis at all, despite being at risk for VTE [6]. It also showed that 50–70% of thromboembolic events occurred in medical patients [7][8]. It is estimated that 1 out of every 6 VTEs can be avoided with adequate TP [9].

The formal recommendation for TP in the 8^th^ consensus of the American College of Chest Physcians, published in CHEST (ACCP 2008), states that every institution should have a formal strategy for active prevention of VTE [1]. These strategies should include all measures that have shown to be effective in improving TP, including periodic monitoring and internal audits of the effectiveness of TP [10].

The primary objective of the study was to determine the proportion of hospitalized patients who are prescribed adequate prophylaxis for VTE in accordance to local institutional guidelines. The secondary objective was to determine the proportion of patients at risk for VTE during their hospital stay who received some form of prophylaxis as recommended by the ACCP 2008 international guidelines.

## Materials and methods

The Trombo-Brit study is a cross-sectional study undertaken at the Hospital Británico in Buenos Aires. Our institution is a high-complexity acute-care general hospital, with 250 beds in its admission area, 176 of which are for general adult hospitalized patients. VTE is performed on preprinted medical orders with a special section in which the attending physician must select options for any of the following modalities: no prophylaxis, graduated compression elastic stockings (ES), intermittent pneumatic limb compression (IPC), or pharmacological prophylaxis. In case that pharmacological prophylaxis is selected, the name of the drug must be stated as well as its dose, route and frequency of administration.

The institutional recommendations for VTE prophylaxis are based on ACCP recommendations and were developed by a steering committee in the year 2001, and updated before the survey was conducted [11]. They are available for consultation in every hospital ward. The guidelines were developed to be used in patients over the age of 18, admitted to the hospital’s general wards and special care units (coronary care unit, intensive care unit and cardiovascular surgery recovery areas). According to the presence and combination of diverse risk factors, the patients are stratified in four risk groups: low, moderate, high and very high risk. For each group, a first line strategy is recommended and other options are suggested (see Additional file 1: Annex 1 for a summary version of the guidelines).

### Population included in the study

Patients that met the following inclusion criteria were included: 1) patients over the age of 18 years old; 2) admission to general wards or to any of the special care units; 3) more than 24 h of admission. Patients excluded from the study were those admitted to certain areas like Maternity or Neonatology, those who had less than 24 h of hospitalization, and patients who were receiving anticoagulant therapy for conditions such as atrial fibrillation, prosthetic heart valves, venous thromboembolism, etc.

### Procedures

The data was collected by physicians of the Internal Medicine and Hematology departments, with previous instructions on how to fill in the case report forms. Each ward was evaluated by a different physician than the one in charge of the ward and without previously alerting to the attending physicians.

A case report form (CRF) for data collection was developed by the authors to estimate the adequacy of the prophylaxis prescriptions according to the institutional guidelines for TP. In order to obtain the TP adequacy rate according to the ACCP 2008 recommendations, the CRF was adapted to the one used in the ENDORSE study [6], which was provided by its principal investigator in a personal communication.

Demographic general data (age, sex, height, weight, day of admission) were collected, as were the reason of clinical admission (medical patients) and type of surgery (surgical patients). Clinical conditions associated to a higher risk of bleeding were recorded as were contraindications to anticoagulation such as low platelet count, kidney failure, and presence of active bleeding or of preexisting coagulopathy. For surgical patients, the type of surgery and anesthesia employed, duration of surgery, its cause and associated risk factors for VTE were assessed. Also, information on the VTE prophylaxis used was obtained: the drug prescribed and its dosage, date of commencement, and the indication of mechanical measures for TP. In the cases where mechanical measures were indicated for TP, the investigators were instructed to verified the properly use of them. For the analysis, patients who met the inclusion and exclusion criteria were stratified in four risk categories for the development of VTE: low, moderate, high and very high risk of DVT according to the presence of different risk factors as detailed in the local guidelines. TP prescriptions were considered as “adequate” if the medical prescription coincided with one of the options for prophylaxis listed in the guidelines; “deficient” if patients had not received TP or if it was less than the recommended by the guidelines and “excessive”, when patients received more TP than the recommended one or if TP was prescribed in situations where it was considered unnecessary. A global adherence rate was obtained by dividing the number of total adequate indications over number of total indications.

Contraindications to pharmacological prophylaxis were defined as at least one of the following conditions: a platelet count less than 50.000/mm3, active bleeding or suspicion thereof, recent intracranial hemorrhage (< 7 days), known bleeding disorder or severe hepatic failure.

Data of renal function, height and body weight were collected to calculate the creatinine clearance by Cockcroft’s method and body mass index (BMI). The serum creatinine level closest to the date of inclusion was recorded. To evaluate the secondary objective (adequacy of the prescriptions to the recommendations of the ACCP guidelines), patients at risk for VTE were stratified in accordance to ACCP 2008 guidelines (see Additional file 2: Annex 2, Tables 1 and 2) [1]. Medical patients were stratified in two groups: patients at risk for VTE and patients with no risk. Surgical patients were grouped in four risk categories: low, moderate, high and very high of DVT. The global adherence rate was determined by the quotient of total adequate indications over total indications performed in all the patients at risk for VTE.

### Statistical analysis

A sample size of 264 patients was calculated based on an estimate of 50% of adherence rate as reported in studies that included Latin American countries [6][12] (80% potency and an alpha error of 0.05, accepted error 11%). It was estimated that it would be necessary to enroll at least 350 patients since it was estimated that approximately 30% of them would not meet the pre-specified inclusion criteria. To achieve the required sample size, three surveys were performed separated by an interval of 25 days each.

The data were analyzed using the statistical software Stata® 10.1 and Microsoft® Excel® 2007. Categorical variables were summarized by frequency and percentage. Quantitative variables were described using mean (standard deviation, SD) or median (interquartile range, IQR) according to whether they had symmetrical or asymmetrical distribution respectively. Because the study design included the realization of the overall survey at three different times, we foresaw the possibility of occurrence of a “learning effect” by which the adherence of the indications to the guidelines would improve with systematic monitoring [10]. For this reason we developed a logistic regression model to assess independent factors associated with non-adherence to recommendations of institutional TP adjusted for the time of survey (periods 1, 2 and 3). The following variables were included in the univariate analysis: age, sex, BMI over 30, cancer, type of admission (medical or surgical), area of admission (general ward or special care units), lower limb immobility, presence of infection, contraindications to pharmacological prophylaxis, history of chronic obstructive lung disease or respiratory failure, and current respiratory infection. Variables that reached a p value < 0.2 in the univariate analysis were incorporated into the initial model in a sequential fashion, and remained in the model if they reached a p value < 0.05 in the Wald test, or if they produced significative changes in the coefficient of another variable already included in the model. Model calibration was evaluated by the Homer-Lemeshow “goodness of fit” statistical test. Analysis of area under the ROC curve (receiver operator characteristic analysis) was planned to determine whether the model adequately discriminated subjects with and without the event.

### Ethical considerations

The study protocol was designed by the two principal authors (FJB and JMC), and approved by the Hospital Británico’s Institutional Review Board (IRB). Due to the fact that the study only involved collection of non sensitive data, the IRB granted the investigators an exception to the obtaining of an informed consent from each patient.

## Results

**Figure 1:**
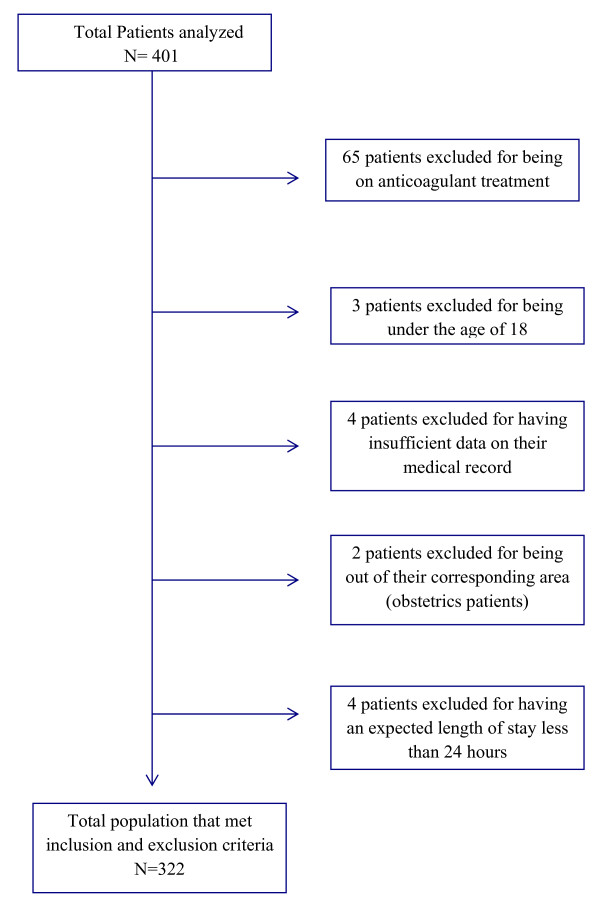
Flow chart of patients enrolled in the study.

Between July and September 2010, the three planned surveys were performed. A total of 409 patients were analyzed, of which 78.7% (n = 322) were eligible in accordance to the study criteria (see Figure 1). Eighty seven patients (21.3%) were excluded from analysis because of the presence of either one of the predefined exclusion criteria (66 patients were on anticoagulant therapy, three patients under 18 years old and two obstetric patients that were admitted in a general ward, four patients had expected hospital stay of less than 24 h, and twelve patients had insufficient data in their medical record for the survey).

Of the final analysis population (n = 322), 219 belonged to the medical group (68%), and 103 (32%) to the surgical group. The clinical characteristics of each group are summarized in Table 1. The overall median age was 63 years old (IQR 54–76) and 45% of patients (n = 144) were women. The overall median length of hospitalization until the survey was 5 days (IQR 2–13). The median length of hospitalization up to the survey was 4 (IQR 1–9) and 6 days (IQR 3–14) for the surgical and medical groups respectively.

**Table 1:** Summary of clinical characteristics of included patients

		**Total N=322**	**Surgical N=103 (32%)**	**Medical N=219 (68%)**
**Female Sex, n (%)**		144 (44.7)	40 (38.8)	104 (47.5)
**Age (years) †**		63 ± 18.42	61.13 ±18.9	63.8 ±18.2
**BMI (kg/m**^**2**^**) †**		25.9 ± 5.31	26.5 ±5.6	25.6 ±5.17
**Length of stay up survey,** **median (IQR)**		5 (2 - 13)	4 (1 - 9)	6 (3 - 14)
**Renal failure (ClCreat<30), n (%)**		15 (4.75)	1 (1.01)	14 (6.45)
**Risk for developing VTE, n (%)**	Low	10 (3.11)	4 (3.9)	6 (2.74)
	Moderate	26 (8.07)	7 (6.8)	19 (8.68)
	High	228 (70.81)	38 (36.9)	190 (86.76)
	Very high	58 (18)	54 (52.4)	4 (1.83)

BMI: body mass index, ClCreat: creatinine clearence (Cockcroft), IQR: interquartile range, VTE: venous thromboembolism.

† Mean ± standard deviation.

According to the criteria for risk stratification of our institutional guidelines more than 85% of patients (n = 286) belonged to the high and the very high risk groups of developing VTE (89.3% and 88.5% for the surgical and medical group respectively). Only 36 patients (11.2%) were considered at low or moderate risk of developing venous thrombosis.

Approximately one third of the patients (31.7%, n = 102) had cancer and 72.6% (n = 234) had immobilization, defined as confinement to bed or to a chair for at least 50% of the day. Near 20% of surgical and 14.2% of medical patients were obese (total 15.6%, BMI greater than 30 kg/m^2^)(see Table 2). Another frequent risk factor identified was chronic obstructive pulmonary disease (COPD) presenting in 10.5% (n = 34) of patients.

**Table 2:** **Preexisting risk factors for developing VTE**^**‡**^

	**Total N=322 n (%)**	**Medical N=219 n (%)**	**Surgical n=103 n (%)**
**History of VTE**	1 (0.31)	1 (0,46)	0
**Heart failure**	10 (3.11)	9 (4,11)	1 (0,97)
**COPD**	34 (10.56)	28 (12,8)	6 (5,83)
**Lower limb venous insufficiency**	14 (4.35)	8 (3,65)	6 (5,83)
**BMI > 30 kg/m**^**2**^	50 (15.6)	31 (14,22)	19 (18,45)
**Cancer †**	102 (31.7)	61 (27,8)	41 (39,8)
**Lower limb immobility §**	67 (20.8)	57 (26)	10 (9,7)
**Complete rest for over 72 hs ¥**	167 (51.8)	124 (56.6)	43 (41.7)

VTE: venous thomboembolism, COPD: chronic obstructive pulmonary disease, BMI: body mass index.

† Active cancer, cancer surgery or cancer treatment at present.

§ Immobility prior to admission due to lower limb paresis or paralysis.

¥ Reduced mobility (rest in bed or a chair for over 50% of the time, and not moving further than the toilet).

‡ The frequency of risk factors surpasses the total number of patients, as some subjects had more than one risk factor. No patient with inherited prothrombotic states were found in the analyzed group. One patient in the anticoagulated group had a history of a prothrombotic condition.

Reasons for admission in both groups are described in Tables 3 and 4.

**Table 3:** Type of surgery, number and percentage of patients with adequate prophylaxis for VTE

	**Surgical (n/%)**	**Adequate prophylaxis**
**Major abdominal**	8 (7.8%)	6 (75%)
**Head and neck**	5 (4.9%)	5 (100%)
**Cardiovascular**	10 (9.8%)	7 (70%)
**Minor abdominal**	4 (3.9%)	2 (50%)
**Colorectal**	11 (10.8%)	8 (72.7%)
**Gynecological**	5 (4.9%)	4 (80%)
**Hepato-biliary**	2 (1.9%)	0
**Neurosurgery**	5 (4.9%)	4 (80%)
**Orthopedic surgery other than THR, TKR and HFS**	3 (2.9%)	3 (100%)
**Major Orthopedic surgery (includes THR, TKR and HFS)**	36 (34.3%)	30 (83.3%)
**Thorax**	6 (5.9%)	4 (66.7%)
**Urologic**	8 (7.8%)	6 (75%)
**TOTAL**	103	79 (76.7%)

THR: total hip replacement; TKR: total knee replacement; HFS: hip fractures surgery.

**Table 4:** Causes for admission and/or clinical risk conditions for VTE in medical patients †

	**Medical pts. (n/%)**	**Adequate prophylaxis**
**Congestive heart failure**	20 (9.1%)	16 (80%)
**Non-respiratory infection**	66 (30.1%)	48 (72.7%)
**CVA (ischemic and hemorrhagic)**	15 (6.8%)	10 (66.7%)
**Acute respiratory failure (COPD exacerbation, pneumonia or other etiology)**	64 (29.2%)	50 (78%)
**Multiple Trauma**	3 (1.37%)	1 (33%)
**Acute inflammatory and/or rheumatologic condition.**	12 (5.5%)	9 (75%)
**AMI (including acute coronary syndromes)**	11 (5%)	9 (81.8%)
**Non-surgical cancer**	61 (27.8%)	42 (68.8%)
**TOTAL**	n: 219	154 (70.3%)

† The sum of risk factors can surpass the total number of patients due to more than one risk factor present.

VTE: venous thromboembolism, CVA: cerebrovascular accident, COPD: chronic obstructive pulmonary disease, AMI: acute myocardial infarction.

The overall TP adherence rate according to institutional guidelines was 72.7% (95% CI 63 – 80.5%). Adequate TP in the high and very high risk groups was found in 79% of patients (227/284, 95% CI 71.6 – 87.2%), whilst in the low risk group, antithrombotic prevention was found to be adequate in 50% of the patients. In contrast, only 7.7% of the patients evaluated in the moderate risk group had an adequate TP (Table 5). In these two last groups (low and moderate risk) we observed that in most cases the TP was inadequate due to an excessive prescribed dose of low molecular weight heparin (LMWH)(25/36 patients, 69.4%), and in only 14% of them (5/36) inadequacy was due to no TP at all. However, as the patients at low and moderate risk for VTE admitted to hospital represented only 11% of the total population, the weight of this incorrect prophylaxis did not have a significant impact on the overall adequate TP rate.

**Table 5:** Summary of TP indications according to risk group

**Risk Group**	**Adequate N (Group %)**	**Inadequate (deficit) N (Group %)**	**Inadequate (excess) ‡ N (Group %)**	**Total (% of total patients)**
**Low**	5 (50)	0 (0)	5 (50)	10 (3.11)
**Moderate**	2 (7.69)	5 (19.23)	19 (73.1)	26 (8.07)
**High**	179 (78.5)	44 (19.3)	5 (2.2)‡	228 (70.81)
**Very High**	48 (83)	8 (13.8)	2 (3.45)‡	58 (18)
**TOTAL**	234 (72.67)	57 (17.7)	31 (9.63)	322 (100)

TP: thromboprophylaxis.

‡ Inadequate due to excess, since the dose prescribed for pharmacological prophylaxis was higher than that suggested, even adjusted for body mass index.

The proportion of adequate TP in the surgical group in accordance to local guidelines was 76.7% (95% CI 68.5 – 85%). Patients who had an orthopedic major intervention (n = 39, 38% of the patients in the surgical group) had an 84.6% adequate TP rate (33/39). This group of surgical patients had the highest adherence level for DVT prophylaxis. Adequate TP rate in the medical group was 70.3% (95% CI 60 – 78%).

About 20% of admitted patients belonging to high and very high risk categories received inadequate TP (59/286), being deficient in 88% of cases (52/59). In analyzing the reason for this deficient TP we found that half (26/52, 50%) of them were patients who had contraindications for pharmacological prophylaxis, and mechanical measures (IPC/elastic stockings) for TP were not prescribed or were not implemented.

Table 6 shows patients with a contraindication for pharmacological prophylaxis, and those who had received unfractionated heparin (UFH) due to severe renal failure. A 5% of all patients had severe renal failure (calculated creatinine clearance less than 30 ml/min) and most of them belonged to the medical group (14 of 15 patients). Among the causes of contraindication for the use of antithrombotic drugs, 9 patients (2.78%) had active bleeding and 21 patients (6.5%) had severe thrombocytopenia (less than 50,000 platelets/mm³). Though the distribution of patients with contraindications for pharmacologic TP was even among the surgical and medical groups, 88% of them (31/35) were in the high risk category of DVT. Of all patients with contraindication for the use of antithrombotic drugs, only a 20% received correct TP (6/35). This makes these patients a special interest group because of their high risk for VTE, and the large number of errors due to the scant use of mechanical measures.

**Table 6:** Contraindications to pharmacological TP or heparin dosage adjustment necessity †

	**Total (n=322)**	**Medical (n=219)**	**Surgical (n=103)**
**Severe renal failure (ClCreat ≤ 30 ml/min) ¥**	15 (4.7%)	14 (6.45%)	1 (1.01%)
**Thrombocytopenia (<50000/mm**^**3**^**)**	21 (6.52%)	21 (9.59%)	0
**CNS hemorrhage in previous 7 days**	4 (1.24%)	4 (1.83%)	0
**Severe liver failure**	7 (2.17%)	6 (2.74%)	1 (0.97%)
**Suspected or active bleeding**	9 (2.8%)	9 (4.11%)	0
**Known bleeding diathesis**	3 (0.93%)	2 (0.91%)	1 (0.97%)
**TOTAL patients with contraindications**	35 (11%)	32 (14.6)	3 (2.9)

TP: thromboprophylaxis, ClCreat: creatinine clearence, CNS: central nervous system.

† Some patients had more than one risk factor for bleeding.

¥ Adjustments required and/or change of drug. Calculations made on 316 patients (n=217 medical group and n=99 in the surgical group).

Pharmacologic prophylaxis was the most frequent method used for DVT prevention in near 80% of the patients (n = 232, 78%) and its use was slightly less frequent in medical patients (n = 164, 75%) than surgical patients. Most patients with pharmacologic TP received LMWH as drug of choice (Table 7). Mechanical prophylaxis was chosen in 7.5% of cases and was used primarily in patients with a contraindication to receive heparin or as a complement to pharmacological TP. The use of mechanical devices for TP was very erratic and only 50% of patients with a prescription for the use of this method actually had the device fitted (12/24 patients overall, 4/8 for elastic stockings and 8/16 for IPC).

**Table 7:** Summary of VTE prophylaxis measures used

	**Total n=322**	**Medical n=219**	**Surgical n=103**
**A) Mechanical prophylaxis:**	**24 (7.5%)**	**17 (7.7%)**	**7 (6.8%)**
**Graduate-compression elastic stockings**	8 (2.5%)	6 (2.7%)	2 (2%)
**Pneumatic intermittent compression**	16 (5%)	11 (5%)	`5 (4.8%)
**B) Pharmacological prophylaxis:**	**252 (78%)**	**164 (75%)**	**88 (85%)**
**Low molecular weight heparin**	211 (84%)	130 (79.%)	81 (92%)
**Unfractionated heparin**	40 (16%)	33 (20%)	7 (7.95%)
**Fondaparinux**	1 (0.4%)	1 (0.61%)	0

VTE: venous thromboembolism.

### Antithrombotic prophylaxis according to the ACCP 2008 international guidelines

In evaluating the adherence TP rate according to the ACCP 2008 International Guidelines we found that out of 272 evaluable patients, 235 met the standards of adequate prophylaxis (86.3%, 95% CI 79.4 – 89.5%). In the medical patient’s group the adequate TP rate was 83.3% (95% CI 79.4 – 92.1%), whilst in surgical patients (101 evaluable patients according ACCP guidelines) this rate was 92% (95% CI 79.6 – 96.4%).

### Analysis of the surveys periods

There was a statistically significant progressive increase in the adequate TP rate among the three periods. In period 1, 66.1% of the prescriptions were adequate, while in periods 2 and 3 were 71.1% and 81.5% respectively (chi for trend 6.45 p = 0.011). In the logistic regression model we found that the presence of contraindication for pharmacological TP (OR 28, 95% CI 10.6 – 75) and belong to low and moderate risk categories (OR 25.2, 95% CI 10 – 64) were independent risk factors for poor adherence to the TP guidelines. The final model showed adequate calibration and discrimination.

## Discussion

Antithrombotic prevention was adequate in 72% of admitted patients according to the Hospital Británico’s institutional guidelines for TP. In 10% of patients prevention was inadequate due to “excess” of thromboprophylaxis (31/322), and in 18% due to “deficit” in prescribing. If we use the 8^th^ ACCP International Consensus Guidelines as a comparator, the percentage of patients receiving adequate prophylaxis was 86%. In a comparable study (ENDORSE) where the same methods were used, the adequate TP rate in hospitalized patients in the 4 participating Latin American countries was less than 50% [6].

A 70% of patients in the medical group received adequate prophylaxis. If we add to this group the patients whom received prophylaxis higher than recommended (excessive TP), we would find that 90.4% of patients received some form of prevention of VTE. Nevertheless, one out of every five high-risk medical patients (44/194) did not receive any TP, including patients with stroke (66% adequate TP rate), and patients with active cancer (68% adequate TP rate). Two thirds of the high and very high-risk medical patients who had inadequate TP by deficient prescription, also had contraindications for pharmacological prophylaxis (25/38, 65.8%). In other words, more than half of the errors of TP in high-risk patients were due to a failure in prescription or because of a failure in implementing mechanical measures (IPC/ES). It is notable that of 35 patients in whom pharmacological prophylaxis was contraindicated, only 6 (17%) had adequate TP and in 28 patients (80%) it was inadequate due to absence of any prophylaxis. Moreover, even when mechanical measures were prescribed by the attending physician, 50% of cases they were not carried out because the device was not available.

Only 36 of the 322 patients had low or moderate risk for VTE. This probably was due to the fact that the Hospital is an acute care institution, where most patients have multiple risk factors for VTE.

Given the above, it is not surprising that the analysis of the logistic regression model identified both the presence of a contraindication to pharmacological TP and belonging to the groups of low and moderate risk, as independent factors associated to poor adherence to the local guidelines. Although the confidence interval of this association is very wide, and this may be due to the limited number of patients with these characteristics, the lower limit of the confidence interval for the OR obtained in both cases is 10. This means that, in the best case, the presence of either of these two factors multiplies 10 times the chance for inadequate TP prescription.

The fact that the majority of patients at the Hospital were in the high risk category for VTE, simplifies the prescription of antithrombotic prophylaxis, due to the lower need for indicating different doses of antithrombotic agents and the widely use in our institution of LMWH in moderate doses.

Some patients at moderate risk for VTE should have received a lower dose of LMWH, a fact not always taken into account by the attending physician, increasing the errors due to an excess of prescription. In fact, one out of ten patients received a higher dose of heparin than the recommended according to their risk and this could potentially expose them to a higher incidence of bleeding.

### Study limitations

Being a cross-sectional study, as all of this type, we could not know the incidence of VTE of the population surveyed. In addition, as the study was performed in three different periods, there could have been a “learning effect” in the attending physicians. This showed up in the statistical analysis, as there was a significant difference in the adherence rate in the 3 different periods. Due to the limited number of patients, it was not possible to evaluate this effect in depth.

Though the Hospital Británico’s institutional guidelines for TP has not been validated prospectively to determine the association between its risk category assigned and the development of VTE, it is noteworthy that the literature is scarce in studies of these characteristics. In fact only a few RAMs (risk assessment methods) have been validated, and these were retrospective validations and in special populations [13,14].

## Conclusions

In conclusion we can say that while the adequate TP rate in our hospital was higher than those reported in countries in our region, there are still some areas where there is much to improve. We should pay special attention into two different areas: those patients with a contraindication to use pharmacological antithrombotic agents, especially those who require mechanical measures to prevent VTE, and patients with a low or moderate risk of DVT. The use of systematic audits will reveal in the future new areas in which adjustments will be required.

## Competing interest

The authors of this study declare not having any conflicts of interest.

## Authors’ contributions

FJB and JMC were involved in the design of the study, analysis of data and in writing the manuscript. FJB, NE, DP, VP, GP, LF, MEO, SP, SP were involved in data acquisition. FJB perform the staistical analysis. JB and EB revised the manuscript. JE has collaborated in the english translation of the manuscript. All authors read and approved the final manuscript.

## Supplementary Material

Additional file 1 **Annex 1.** (Next two pages): Summarized institutional thromboprophylaxis guidelines. Click here for file

Additional file 2 **Annex 2.** Table 1 ACCP 2008 Guidelines: medical patients at risk for VTE and suggested prophylaxis. Table 2 ACCP 2008 Guidelines: surgical patients at risk for VTE and suggested prophylaxis. Click here for file
