# Supplementary material for: Cross-sectional study of adherence to venous thromboembolism prophylaxis guidelines in hospitalized patients. The Trombo-Brit study
Source: Thromb J. 2012 May 18;10:7. doi: 10.1186/1477-9560-10-7 (PMC3517353; doi:10.1186/1477-9560-10-7)
Supplement: Additional file 1 — Annex 1. (Next two pages): Summarized institutional thromboprophylaxis guidelines. [file 1477-9560-10-7-S1.doc]

| **Annex 1** (Next two pages): Summarized institutional thromboprophylaxis guidelines | | | | | | | |
| --- | --- | --- | --- | --- | --- | --- | --- |
|  |  | | DVT Prophylaxis Guide for SURGICAL ADMISSIONS | | | | |
| Age | Durationof surgery | | Presence of riskfactors | | DVT risk group | APPROACH | |
| 18-39years | Any duration | | NO | | LOW | Early deambulationGraduated elastic stockings | |
|  |  |  | Yes(group 1) | | MODERATE | IPCUFH 5000 U BIDLMWH: Enoxaparin 20 mg OD | |
|  |  |  | Yes(group 2) | | HIGH | IPCUFH 5000 U TIDLMWH: Enoxaparin 40mg OD | |
| ≥ 40years | < 45’ orminor surgical procedures (arthroscopy, laparoscopic surgery, spinal or transurethral surgery) | | NO | | LOW | LOW RISK approach | |
|  |  |  | Yes(group 1) | | MODERATE | MODERATE RISK approach | |
|  |  |  | Yes(group 2) | | HIGH | HIGH RISK approach | |
|  | > 45’ | | | | HIGH | HIGH RISK approach | |
| Patients who have any risk factors of group 3 independent of age or duration of surgery | | | | | VERY HIGH | IPC or Graduated elastic stockingsPLUSLMWH: Enoxaparin 40 mg OD | |
| Risk groups for VTE | | | | | | | |
| LOW RISK | | (1) MODERATE RISK | | (2) HIGH RISK | | | (3) VERY HIGH RISK |
| No risk factors for VTE | | -Oral contraceptives / Estrogen therapy- Chronic Venous Insufficiency- Pregnancy and postpartum (6 weeks)- Airplane trip (more than 8 hours of flight in the last month)-Trauma/abdominal surgery in the previous six weeks-Acute inflammatory or infectious disease-Rheumatological disease | | - Chronic respiratory failure- History of congestive heart failure/COPD- Obesity (BMI > 30)- Inflammatory bowel disease- Myeloproliferative syndromes- Cancer (active) or cancer therapy- Nephrotic syndrome- Extensive Burns- Recent myocardial infarction or acute coronary syndrome- Critical care patients- Sepsis | | | -Multiple trauma-History of recent stroke-Lower limb immobility-Trombophilia-History of DVT/PE-Age >60 years with multiple risk factor for VTEType of surgery:Bricker / pancreaticduodenectomyPelvic exenteration / Radical vulvectomyTHR and TKR-Hip fracture surgeryBariatric surgery |
| IPC: intermittent pneumatic limb compression, LMWH: low molecular weight heparin, UFH: unfractioned heparin, TKR: total knee replacement, THR: total hip replacement.VTE: venous thromboembolism, BMI: body mass index, DVT: deep venous thrombosis, PE: pulmonary embolismBold indicates the behavior suggested as first-line prophylaxis for VTE. | | | | | | | |

| DVT Prophylaxis Guide for CLINICAL ADMISSIONS | | | | |
| --- | --- | --- | --- | --- |
| Risk factors for VTE | DVT RISKGROUP | | APPROACH | |
| Any Age without Risk factors | | LOW | | - Early Ambulation- Elastic stockings |
| -Age > 40 plus poor ambulation (stays in bed or in a chair more than 50% of the day)-Oral contraceptives / Estrogen therapy- Chronic Venous Insufficiency- Pregnancy and postpartum (6 weeks)- Airplane trip (>8 hours of flight in the last month)-Trauma/abdominal surgery in the previous 6 weeks-Acute inflammatory or infectious disease-Rheumatological disease | | MODERATE | | - Elastic stockings- IPC- LDUH 5000 U sc bid- LMWH: Enoxaparin 40 mg OD |
| - Respiratory failure/decompensated COPD/Pneumonia- Cardiac Failure-History of Cardiac Failure/COPD- Obesity (BMI > 30)- Inflammatory bowel disease- Myeloproliferative syndromes- Cancer (active) – Cancer therapy- Nephrotic syndrome (active)- Extensive Burns- Myocardial infarction/Acute coronary syndrome- Critical care patients- Sepsis- Stroke with lower-extremity paresis- Lower limb immobility | | HIGH | | - IPC- LDUH 5000 U bid- LMWH: Enoxaparin 40 mg OD |
| -Major trauma-Trombophilia- History of previous DVT/PE- Respiratory failure, on mechamical ventilation | | VERY HIGH | | - IPC or Elastic stockingsPLUS- LMWH: Enoxaparin 40 mg OD |
| IPC: intermittent pneumatic limb compression, LMWH: low molecular weight heparin, LDUH: low dose unfractioned heparin, TKR: total knee replacement, THR: total hip replacement.VTE: venous thromboembolism, BMI: body mass index, DVT: deep venous thrombosis, PE: pulmonary embolism | | | | |
