# Supplementary material for: Cross-sectional study of adherence to venous thromboembolism prophylaxis guidelines in hospitalized patients. The Trombo-Brit study
Source: Thromb J. 2012 May 18;10:7. doi: 10.1186/1477-9560-10-7 (PMC3517353; doi:10.1186/1477-9560-10-7)
Supplement: Additional file 2 — Annex 2. Table 1 ACCP 2008 Guidelines: medical patients at risk for VTE and suggested prophylaxis. Table 2 ACCP 2008 Guidelines: surgical patients at risk for VTE and suggested prophylaxis. [file 1477-9560-10-7-S2.doc]

Annex 2

| **Table 1. ACCP 2008 Guidelines: medical patients at risk for VTE and suggested prophylaxis** | |
| --- | --- |
| **Admission due to cardiac failure or non-infectious respiratory disease or pulmonary infection** | UFH or LMWH, or IPC/ES for patients at risk for bleeding |
| **Complete immobilization or immobilization with bathroom privileges associated to one or more of the following factors:**   - **History of VTE** - **Active cancer** - **Treatment for cancer or active malignancy on admission, infection (non-respiratory (non-respiratory)** - **Ischemic or hemorrhagic CVA** - **Inflammatory or rheumatological disease** | UFF o LMWH, or IPC/ES for patients at risk for bleeding |
| **Present admission to ICU/CCU, or assisted mechanical ventilation** | FHF or LMWH, or IPC/for patients at risk for bleeding |
| ACCP: American College of Chest Physicians, VTE: venous thromboembolism, CVA: cerebrovascular accident, ICU: intensive vare unit, CCU: critical care unit, UFH: unfractionated heparin, LMWH: low molecular weight heparin, IPC: intermittent pneumatic compression, ES: elastic stockings | |

| **Table 2. ACCP 2008 Guidelines: surgical patients at risk for VTE and suggested prophylaxis .** | | |
| --- | --- | --- |
| **Very high risk surgery** | - **Total hip replacement** - Total knee replacement - **Hip fracture (includes patients with major trauma)** - Age ≥40 plus history of VTE or active cancer (cancer surgery, ongoing cancer, or cancer therapy on admission) | **LMWH or VKA or Fondaparinux**  LMWH or Fondaparinux o VKA or IPC  **LMWH or Fondaparinux or VKA or IPC/ES + UFH, or IPC/ES for high bleeding risk**  LMWH or fondaparinux or VKA, or IPC/ES + UFH |
| **High risk surgery** | **Patients >60 years, and patients between 40 and 60 with any of the following factors: history de VTE or active cancer (cancer surgery, ongoing cancer or cancer therapy on admission) or a history of inherited prothrombotic states** | LMWH or UFH or IPC/ES |
| **Moderate risk surgery** | Patients between 40 and 60 years, and patients between 18 and 39 years with any of the following factors: history of VTE, or active cancer (cancer surgery, ongoing cancer or cancer therapy on admission) or a history of inherited prothrombotic states. | LMWH or UFH or IPC/ES |
| **Low risk surgery** | **Patients between 18 and 39 without additional risk factors. All surgeries not included in the previous categories.** | Early ambulation |
| ACCP: American College of Chest Physicians, VTE: venous thromboembolism, CVA: cerebrovascular accident, LMWH: low molecular weight heparin, VKA: vitamin K antagonists IPC: intermittent pneumatic compression, ES: elastic stockings, UFH: unfractionated heparin. | | |
